# Neuropeptide S Facilitates Mice Olfactory Function through Activation of Cognate Receptor-Expressing Neurons in the Olfactory Cortex

**DOI:** 10.1371/journal.pone.0062089

**Published:** 2013-04-16

**Authors:** Yu-Feng Shao, Peng Zhao, Chao-Yu Dong, Jing Li, Xiang-Pan Kong, Hai-Liang Wang, Li-Rong Dai, Yi-Ping Hou

**Affiliations:** Departments of Neuroscience, Anatomy, Histology, and Embryology, Key Laboratory of Preclinical Study for New Drugs of Gansu Province, School of Basic Medical Sciences, Lanzhou University, Lanzhou, Gansu, People's Republic of China; University of Medicine and Dentistry of New Jersey, United States of America

## Abstract

Neuropeptide S (NPS) is a newly identified neuromodulator located in the brainstem and regulates various biological functions by selectively activating the NPS receptors (NPSR). High level expression of NPSR mRNA in the olfactory cortex suggests that NPS-NPSR system might be involved in the regulation of olfactory function. The present study was undertaken to investigate the effects of intracerebroventricular (i.c.v.) injection of NPS or co-injection of NPSR antagonist on the olfactory behaviors, food intake, and c-Fos expression in olfactory cortex in mice. In addition, dual-immunofluorescence was employed to identify NPS-induced Fos immunereactive (-ir) neurons that also bear NPSR. NPS (0.1–1 nmol) i.c.v. injection significantly reduced the latency to find the buried food, and increased olfactory differentiation of different odors and the total sniffing time spent in olfactory habituation/dishabituation tasks. NPS facilitated olfactory ability most at the dose of 0.5 nmol, which could be blocked by co-injection of 40 nmol NPSR antagonist [D-Val^5^]NPS. NPS administration dose-dependently inhibited food intake in fasted mice. *Ex-vivo* c-Fos and NPSR immunohistochemistry in the olfactory cortex revealed that, as compared with vehicle-treated mice, NPS markedly enhanced c-Fos expression in the anterior olfactory nucleus (AON), piriform cortex (Pir), ventral tenia tecta (VTT), the anterior cortical amygdaloid nucleus (ACo) and lateral entorhinal cortex (LEnt). The percentage of Fos-ir neurons that also express NPSR were 88.5% and 98.1% in the AON and Pir, respectively. The present findings demonstrated that NPS, via selective activation of the neurons bearing NPSR in the olfactory cortex, facilitates olfactory function in mice.

## Introduction

Neuropeptide S (NPS) is a newly identified neuromodulator located in the brainstem. NPS selectively binds with high affinity to Gs and Gq protein-coupled receptors, identified as GPR 154 previously and now referred to as NPSR, to produce mobilization of intracellular Ca^2+^ and to increase in cAMP levels [1]. NPS precursor mRNA in the rat is expressed in a group of neurons located between the locus ceruleus (LC) and Barrington's nucleus, the principle sensory trigeminal nucleus, and the lateral parabrachial nucleus [1]. In the mouse, NPS precursor mRNA is only expressed in the Kölliker-Fuse nucleus and pericoerulear area of the brainstem [2]. In contrast, NPSR mRNA is found widely distributed in the rat and mouse brain, mainly in the olfactory cortex, cerebral cortex, thalamus, hypothalamus, amygdala, and subculum [1]–[4].

This profile of NPSR mRNA expression suggests involvement of NPS-NPSR system in the regulation of multiple central functions. Actually, activation of NPSR by central administration of NPS enhances locomotor and exploratory activities, and evokes anxiolytic-like effects in mice [1], [5], [6], and promotes wakefulness in rats [1], [7]. NPS is also involved in antinociception [8], [9], fear expression and extinction [10] and memory processes in mice [11], [12], and facilitates relapse to cocaine seeking in rats [13].

NPS-NPSR system is proposed as a newly identified olfactory regulating system involved in regulation of olfactory perception and/or integration of olfactory or pheromonal information [3], because the high levels of NPSR mRNA expression have been found in many regions of olfactory cortex including the anterior olfactory nucleus (AON), piriform cortex (Pir), tenia tecta (TT), and the anterior cortical amygdaloid nucleus (ACo) and lateral entorhinal cortex (LEnt) in mice [2]. These regions of the olfactory cortex directly receive synaptic input from the olfactory bulb [14], [15] and appear to play a crucial role in the translation of features of inhaled molecules into rich, emotion and memory-evoking tinged perceptions called odors [15]. However, how NPS-NPSR system regulates the olfactory behavior is unknown.

The present study was designed to observe the effects of NPS-NPSR system on the olfactory function in mice following intracerebroventricular (i.c.v.) injections. Olfactory abilities in mice were gauged using the buried food test (to assess the ability of detecting volatile odors) and olfactory habituation/dishabituation test (to assess the ability of detecting and distinguishing the same and different odors). Food intake test was used to clarify the relationship between olfaction and ingestion in mice after i.c.v. administration of NPS. To further identify potential neuronal targets of NPS in the olfactory cortex, NPS-induced Fos immunereactive (-ir) neurons were analyzed using *ex vivo* immunohistochemistry, and the presence of NPSR in these neurons was examined using dual-immunofluorescence microscopy.

## Materials and Methods

### Animals and surgical implantation

Adult male C57BL/6J mice (6 weeks old), were purchased from Experimental Animal Central of Lanzhou University (Lanzhou, PR China). They were housed in an ambient temperature (22±1°C) with a relative humidity of 50% on an automatically controlled 12:12-h light/dark cycle (lights on 8:00–20:00 h, illumination intensity ≈ 100 lx). Food and water were available *ad libitum* except for the period of food deprivation. Each animal was used only once for between-group comparisons in the buried food test, olfactory habituation and dishabituation test, and food intake test. All animals were cared for, and experiments were conducted in accordance with the European Community guidelines for the use of experimental animals (86/609/EEC). The experimental protocol was approved by the Ethics Committee of Lanzhou University (permit number: SCXK Gan 2009–0004).

Under chloral hydrate anesthesia (350 mg/kg, i.p.), mice were placed in a stereotaxic apparatus. A stainless-steel guide cannula (25 gauge) was stereotaxically implanted above the right lateral ventricle (AP −0.2 mm, ML +1.0 mm, DV −1.4 mm, according to the atlases of Paxinos and Franklin, 2001 [16]) for i.c.v. injection. Cannula was chronically fixed to skull with dental cement. A stainless-steel indwelling stylet (32 gauge) was inserted into the guide cannula to prevent occlusion.

### Drug administrations

NPS (mouse, Ser-Phe-Arg-Asn-Gly-Val-Gly-Ser-Gly-Ala-Lys-Lys-Thr-Ser-Phe-Arg-Arg- Ala-Lys-Gln) and [D-Val^5^]NPS (human, Ser-Phe-Arg-Asn-D-Val-Val-Gly-Thr-Gly-Met-Lys-Lys- Thr-Ser-Phe-Gln-Arg-Ala-Lys-Ser), gifts from Prof. Rui Wang, were synthesized by the Department of Biochemistry and Molecular Biology, School of Life Science, Lanzhou University [7], [9], [17]. Fresh NPS (0.1-1 nmol) and NPS (0.5 nmol) + [D-Val^5^]NPS (20 or 40 nmol) were dissolved in 1 µl saline. The drugs and vehicle (saline) were administered through the planted guide cannula with the flow rate 1 μl/min at 17:00 on the test day.

When an experiment was over, mice were injected i.c.v. with 1 µl methylene blue dye through guide cannula and were decapitated under deep anesthesia with chloral hydrate sodium 5 min later. Brains were removed and frozen. Gross dissection of the brain was used to verify the site of drugs or vehicle administration. Only data from animals with dye dispersion through out the ventricle were used.

### Olfactory behavior tests

#### Buried food test

The buried food test was performed as previously described by Yang and Crawley [18]. Briefly, after 7 days recovery following surgery, the mice were fasted for 32 hours starting from 9:00 h, with water available. On the test day, each mouse received an i.c.v. injection of vehicle, NPS or NPS + [D-Val^5^]NPS and then was placed in a plexiglas test chamber (46 cm L×23.5 cm W×20 cm H) containing 3 cm deep of clean bedding made of freshly sterilized and deodorized wood chips. After acclimating to the environment for 15 min, the mouse was removed from the chamber and the mouse chow pellets (1.5 g, Beijing keaoxieli feedstuff Co. Ltd.) were randomly buried 1 cm beneath the surface of the bedding. Then, the mouse was placed back into the chamber and the latency to find the buried food was measured. The latency was defined as the time from the moment when a mouse was placed into the test chamber to the moment when it uncovered and grasped the food in its forepaws and/or teeth [19]. The test chambers were rinsed with distilled water and dried in air after each test. The bedding was changed before each test. The animals were video-recorded, and scored and analyzed by an investigator blind to the drugs administered.

#### Olfactory habituation and dishabituation test

The olfactory habituation and dishabituation test was performed according to Yang and Crawley's previous description [18]. On the test day, each mouse was placed in a test chamber (26 cm L×12 cm W×12 cm H) containing 1 cm deep bedding of clean, fresh wood chips, and was acclimated to the test condition in which a clean, dry cotton-tipped applicator was inserted 4 cm deep through a hole on the lid for 15 min following an i.c.v. injection of drugs or vehicle. The sniffing behavior was defined as when the mouse was orienting towards the tip of replaced applicator absorbed with distilled water, almond extract (1:100 dilution, Supercook, Leeds, UK) or vanilla extract (1:100 dilution, Supercook, Leeds, UK) with its nose within 2 cm or closer to the tip. The sniffing behavior was recorded for 2 min in each trial. The sequence of replaced applicator was water, water, water, almond, almond, almond, vanilla, vanilla and vanilla, with 1 min interval. Habituation is defined as a progressive decrease in olfactory sniffing time towards a repeated presentation of the same odor stimulus and used to evaluate whether an animal can distinguish the same odor. Dishabituation is defined by a reinstatement of sniffing when a novel odor is presented, and used to assess whether an animal can detect a different odor [20]–[22]. The test chambers were rinsed with distilled water and the bedding was changed before each test. The animals were video-recorded and an experimenter blind to the treatment analyzed the animal behavior.

### Food intake test

It is well-known that olfaction is closely related to food intake in mammals, especially in rodents [23], [24]. To determine whether the change of olfaction following NPS administrations influences food ingestion, the amount of food intake was respectively measured at 0.5, 1, 2, 4 and 24 h after central administration of vehicle (n = 10) or NPS (0.5 or 1 nmol, n = 9 in each group) in mice fasted for 32 h.

### Immunohistochemistry

#### Tissue preparation

One and a half hour after NPS (0.5 nmol, n = 4) or vehicle 1 µl (n = 5) i.c.v. administration, animals were anesthetized with over dose of chloral hydrate (400 mg/kg), and perfused via the ascending aorta with 30 ml saline containing heparin (1 U/ml) followed by 4% paraformaldehyde in 0.1 M phosphate buffer (PB). Brains were removed, post-fixed in the same fixative overnight and immersed in 30% sucrose solution in 0.1 M PB at 4°C for 36 h, and coronally sectioned (30 µm) on a cryostat (CM1900, Leica Micro-systems, Heidelberg, Germany) at −20°C and the sections were collected into 0.01 M sodium phosphate buffer (PBS).

#### Fos immunohistochemistry

The floating sections were rinsed in 0.01 M PBS (pH 7.4), treated 30 min in 0.3% H_2_O_2_ in PBS, and incubated in blocking solution (10% bovine serum in PBS) for 1 h. Then the sections were incubated with a rabbit polyclonal antibody against c-Fos (1∶5,000, sc-253, Santa Cruz Biotechnology, Santa Cruz, CA, USA) diluted in PBS containing 1% bovine serum for 48 h at 4°C on an agitator. After rinsing in PBS, sections were incubated with a biotinylated goat anti-rabbit IgG (1∶1000, AP132B, Millipore, Temecula, CA, USA) then with horseradish peroxidase conjugated streptavidin (1∶2,000, SA202, Millipore, Temecula, CA, USA). Both incubations were carried out on an agitator at 4°C overnight. Following rinsing, the sections were immersed in 0.05 M Tris–HCl buffer, pH 7.6, containing 0.05% 3,3′ diaminobenzidine (DAB), 0.01% H_2_O_2_, and 0.6% nickel ammonium sulfate for 2–5 min at room temperature. Finally, the sections were mounted on gelatin-coated glass slides, processed with counter-staining with neutral red, dried, dehydrated, and covered with a coverslip, using DPX, for light microscopy.

#### Dual-immunofluorescence for Fos and NPSR

These sections were incubated with a mixture solution containing rabbit polyclonal antibody against c-Fos and goat anti-NPSR (1∶1,000, sc-162893, Santa Cruz Biotechnology, Santa Cruz, CA, USA) diluted in PBS containing 1% bovine serum for 48 h at 4°C on an agitator following incubation in 10% bovine serum in PBS. The specificity of the anti-NPSR antibody has been demonstrated in previous studies [25], [26]. After several rinses in PBS, sections were incubated with Alexa Fluor® 488-conjugated affinipure donkey anti-rabbit IgG (1∶200, 711-545-152, Jackson ImmunoResearch Laboratories, Inc., PA, USA) and CyTM 3-conjugated affinipure donkey anti-goat IgG (1∶200, 705-165-147, Jackson ImmunoResearch Laboratories, Inc., PA, USA) for 2 h at 37°C. Finally, sections were mounted on slides, covered with a coverslip, using 90% glycerol in PBS, and observed under a fluorescence microscope.

### Data analysis

#### Cell counting

**Figure 1:**
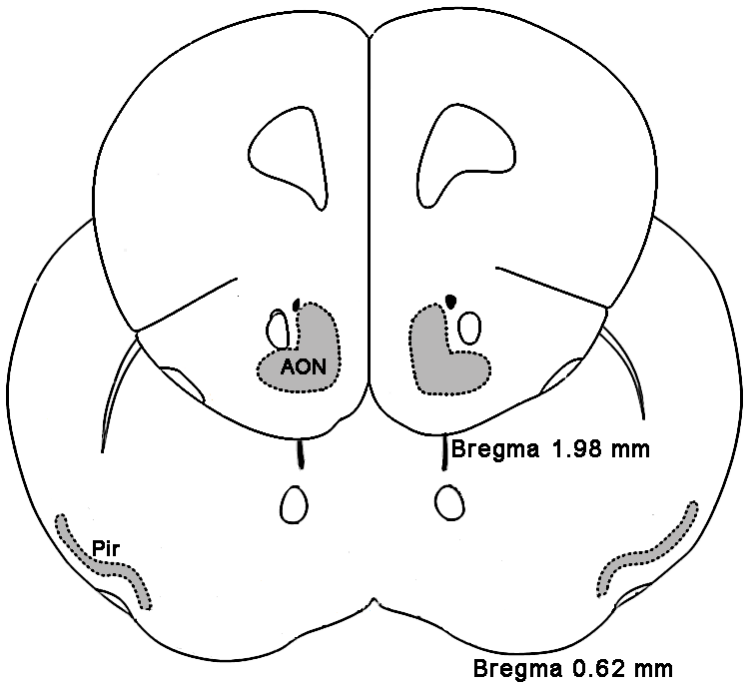
Schematic drawings show the localization of sections used for Fos-ir neurons counting. The grey zones represent the AON (Bregma 1.98 mm) and Pir (Bregma 0.62 mm). Abbreviations: AON, anterior olfactory nucleus; Pir, piriform cortex.

Fos-ir neurons in the AON (Bregma 1.98 mm, Fig. 1), Pir (Bregma 0.62 mm, Fig. 1), VTT (Bregma 2.34 mm), the ACo (Bregma −0.94 mm) and LEnt (Bregma −3.40 mm) were bilaterally counted for each animal treated with NPS or vehicle. The mean value for two sides was calculated.

#### Statistical analysis

The values were expressed as means ± SEM. Data of the buried food test, food intake test and the total sniffing time spent in the olfactory habituation and dishabituation tasks were analyzed using one-way analysis of variance (ANOVA) and post hoc Fisher's least significant difference (LSD) test. Data of olfactory habituation and dishabituation test were analyzed using within-group Repeated Measures ANOVA and followed by the Newman-Keuls tests. The amount of Fos-ir neurons between NPS- and vehicle-treated mice were analyzed using independent student's t-test. In all statistical comparisons, the level of significances was set at *p*<0.05.

## Results

### Effects of NPS on olfactory functions

#### Buried food test

**Figure 2:**
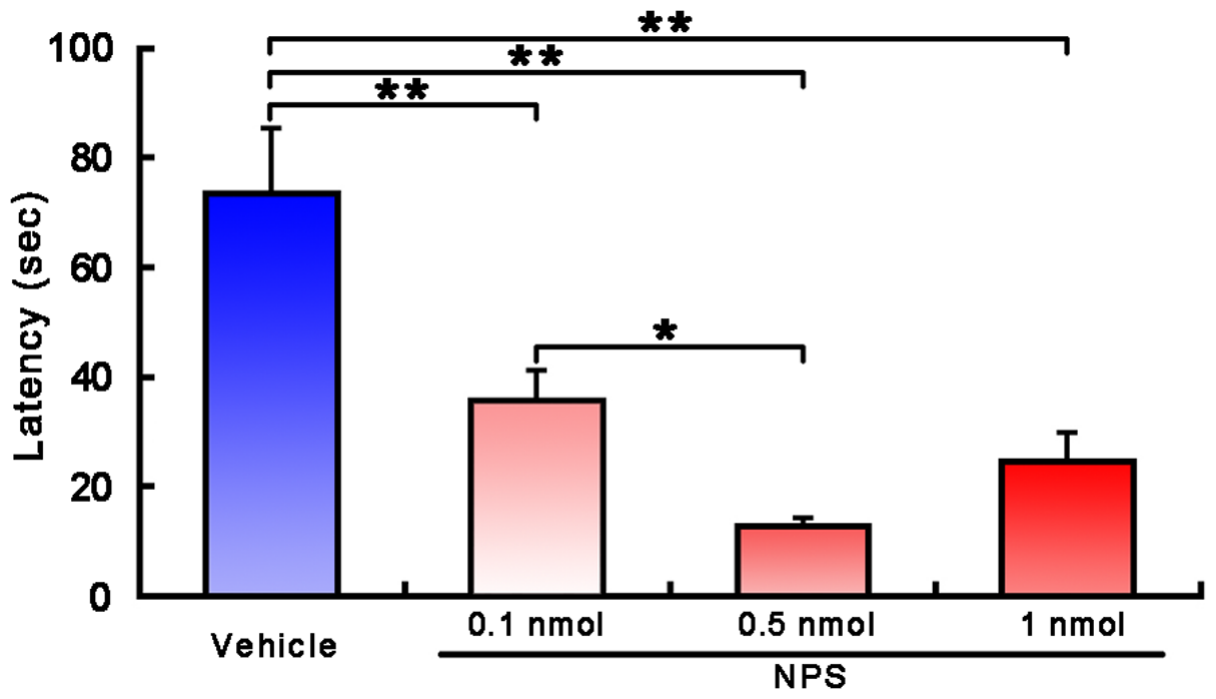
Latency to find the buried food following i.c.v. injection of vehicle or NPS in mice. Values are means ± SEM (n = 10 mice in each group). * *p*<0.05, ** *p*<0.001. Data were analyzed by one-way ANOVA and followed by Fisher's LSD test.

In comparison with vehicle-treated mice, i.c.v. administration of 0.1, 0.5 and 1 nmol of NPS significantly reduced the latency to find the buried food from 73.43±11.77 s to 35.74±5.37 (*p*<0.001), 12.72±1.34 (*p*<0.001) and 24.61±5.04 s (*p*<0.001), respectively (Fig. 2). Among the three doses, 0.5 nmol NPS reduced the latency most (*p*<0.001 and *p*<0.05 compared with vehicle and 0.1 nmol NPS, respectively; Fig. 2). In fact, high dose (1 nmol) of NPS insignificantly reduced the latency as compared to 0.1 nmol NPS (35.74±5.37 s vs. 24.61±5.04 s, *p* = 0.24; Fig. 2).

#### Olfactory habituation and dishabituation test

**Figure 3:**
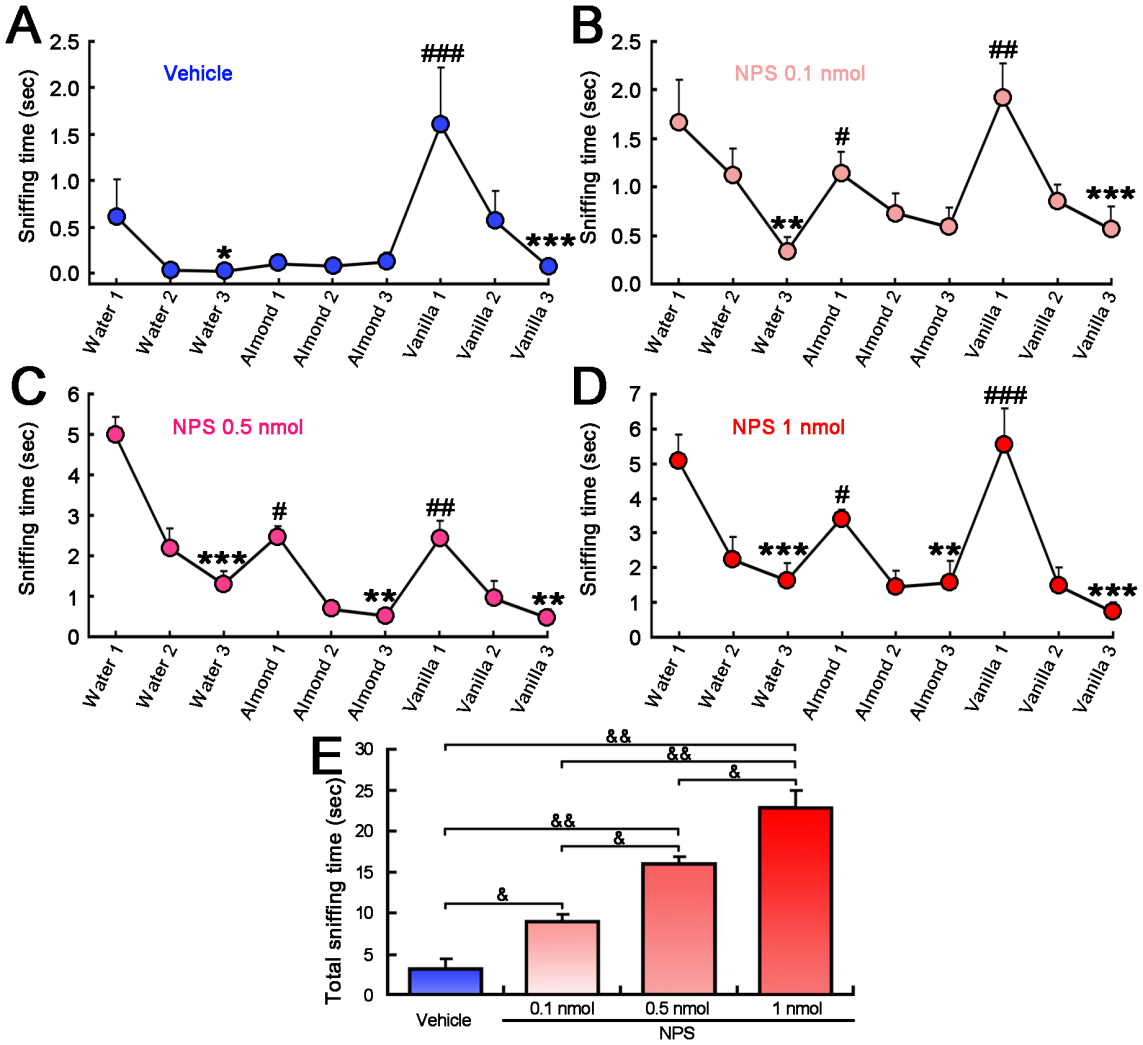
Olfactory habituation and dishabituation test following i.c.v. injection of vehicle or NPS in mice. **A.** Mice treated with vehicle exhibited significant habituation to water, dishabituation almond to vanilla, and habituation to vanilla. **B.** NPS at 0.1 nmol exhibited significant habituation to water, dishabituation water to almond, dishabituation almond to vanilla, and habituation to vanilla. **C.** NPS at 0.5 nmol exhibited significant habituation to water, dishabituation water to almond, habituation to almond, dishabituation almond to vanilla, and habituation to vanilla. **D.** NPS at 1 nmol exhibited significant habituation to water, dishabituation water to almond, habituation to almond, dishabituation almond to vanilla, and habituation to vanilla. **E.** NPS dose-dependently increased the total sniffing time spent in olfactory habituation and dishabituation tasks. Values are means ± SEM (n = 10 mice in each group). * *p*<0.05, ** *p*<0.01, *** *p*<0.001 for habituation;^ #^ *p*<0.05, ^##^ *p*<0.01, ^###^ *p*<0.001 for dishabituation; data were analyzed using within-group Repeated Measures ANOVA and followed by the Newman-Keuls tests. ^&^ *p*<0.01, ^&&^ *p*<0.001; data were analyzed by one-way ANOVA and followed by Fisher's LSD test.

Fig. 3A-D summarize the results from olfactory habitual and dishabitual behavior tests in mice intracerebroventricularly injected with vehicle or NPS (0.1, 0.5 or 1 nmol) to the same and different odors, respectively. Central administration of vehicle induced a habituation to water (*p*<0.05) and vanilla (*p*<0.001), and a dishabituation to vanilla (*p*<0.001, Fig. 3A). Mice administered NPS at 0.1 nmol were able to distinguish almond and vanilla as novel odors, but failed to habituate to the almond odor (Fig. 3B). Relative to the vehicle control, mice habituated and dishabituated all test odors following 0.5 or 1 nmol of NPS administration (Fig. 3C and D), indicating that NPS at these doses could facilitate mice to distinguish all of the same and different test odors. As shown in Fig. 3E, NPS dose-dependently increased the total sniffing time spent in olfactory habituation and dishabituation behavioral tasks.

#### Effect of the NPS on olfactory behavior was blocked by [D-Val^5^]NPS

To identify whether NPSR antagonist blocks the effect of NPS on olfactory abilities, [D-Val^5^]NPS, a selective antagonist of NPSR [27], was injected with or without 0.5 nmol of NPS (i.c.v.) into mice.

**Figure 4:**
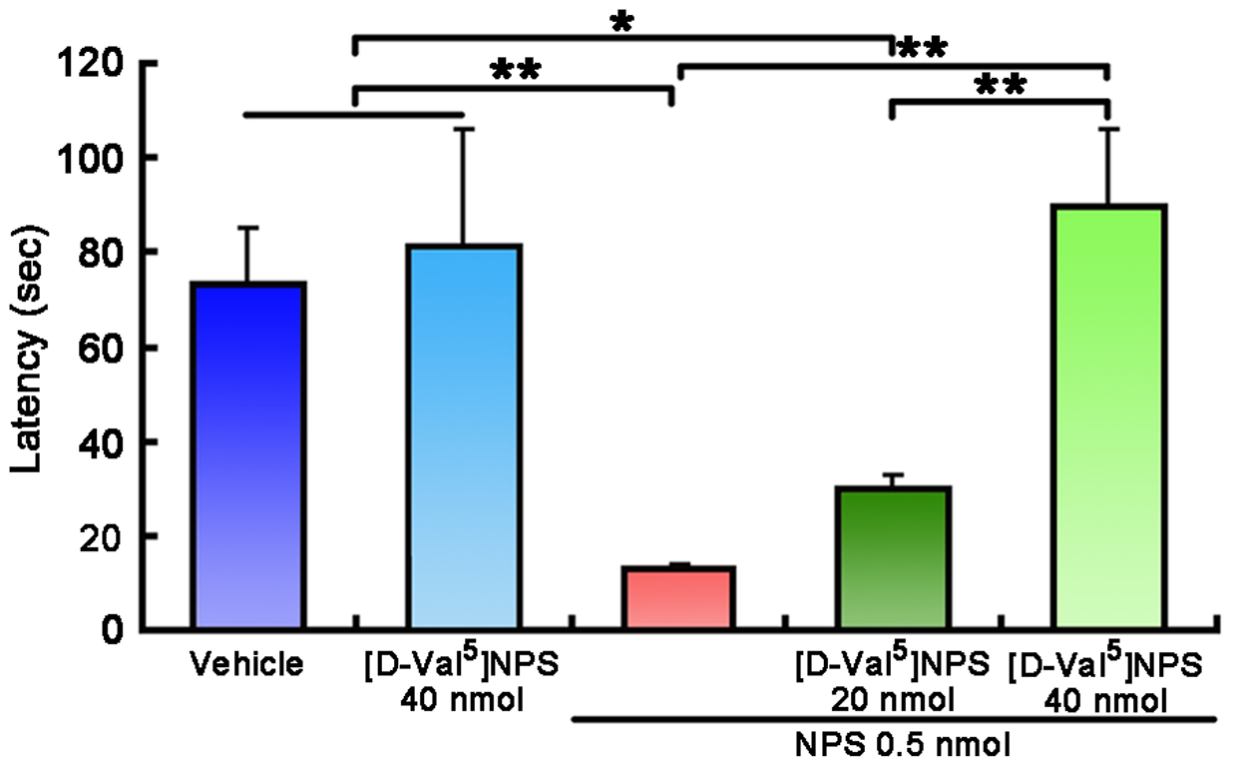
Latency to find the buried food following i.c.v. injection of vehicle, NPS or [D-Val^5^]NPS and NPS + [D-Val^5^]NPS in mice. Values are means ± SEM (n = 10 mice in each group). * *p*<0.05, ** *p*<0.001. Data were analyzed by one-way ANOVA and followed by Fisher's LSD test.

Our results indicated that 40 nmol of [D-Val^5^]NPS significantly antagonized the effect of 0.5 nmol of NPS on the latency to find the buried food (Fig. 4). However, when given alone, 40 nmol of [D-Val^5^]NPS did not affect the latency compared with vehicle (Fig. 4).

**Figure 5:**
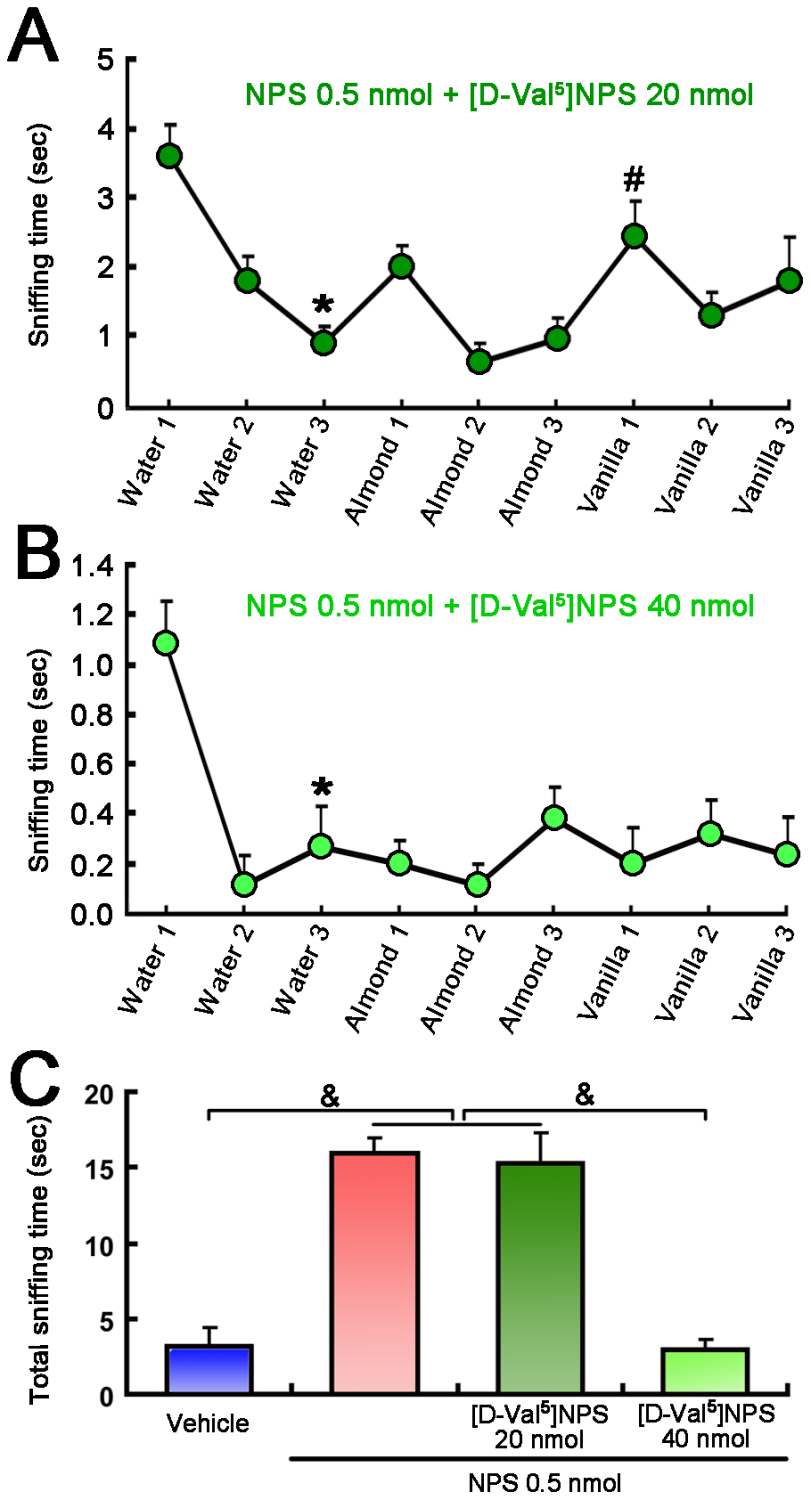
Olfactory habituation and dishabituation test in mice following i.c.v. co-injection of NPS and [D-Val^5^]NPS. **A.** The administration of 0.5 nmol NPS + 20 nmol [D-Val^5^]NPS exhibited significant habituation to water, and dishabituation almod to vanilla. **B.** The administration of 0.5 nmol NPS + 40 nmol [D-Val^5^]NPS exhibited significant habituation only to water. **C.** The administration of 0.5 nmol NPS + 40 nmol [D-Val^5^]NPS significantly blocked the effect of NPS in increase of the total sniffing time in olfactory habituation and dishabituation tasks. Values are means ± SEM (n = 10 mice in each group). * *p*<0.001 for habituation; ^#^ *p*<0.05 for dishabituation; data were analyzed using within-group Repeated Measures ANOVA and followed by the Newman-Keuls tests. ^&^ *p*<0.001, data were analyzed by one-way ANOVA and followed by Fisher's LSD test.

Administration of 20 nmol [D-Val^5^]NPS significantly blocked the effects of 0.5 nmol NPS on olfactory differentiating ability (Fig. 3C) towards water and almond, but not vanilla (Fig. 5A). Further, 40 nmol [D-Val^5^]NPS completely inhibited the effect of NPS on olfactory differentiating behavior (Fig. 5B) and markedly reversed NPS-induced increase in total sniffing time spent in olfactory habituation and dishabituation tasks (Fig. 5C).

### Inhibitory effects of NPS on food intake

**Figure 6:**
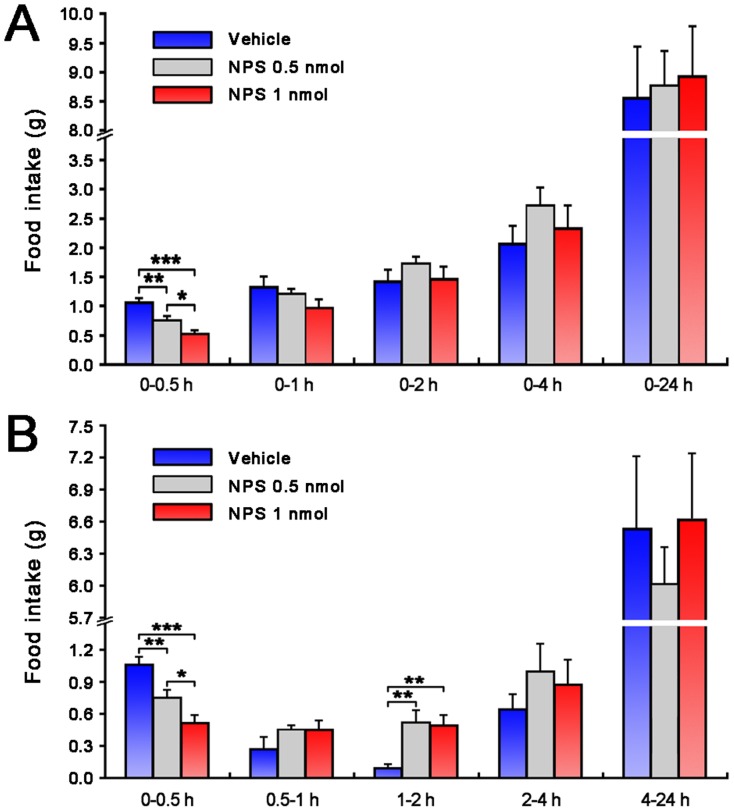
Effects of NPS on cumulated food intake (A) and intake in periods of time (B) in fasted mice. Vehicle, 0.5 or 1 nmol of NPS were i.c.v. administrated in mice fasted for 32 h. Values are means ± SEM (n = 10 mice in vehicle group, n = 9 mice in each NPS group). * *p*<0.05, ** *p*<0.01, *** *p*<0.001. Data were analyzed by one-way ANOVA and followed by Fisher's LSD test.

Fig. 6A and B summarize the effects of NPS (i.c.v.) on cumulative and timed food intake 0.5, 1, 2, 4 and 24 h following treatment in fasted mice. During the first half hour, 0.5 and 1 nmol of NPS dose-dependently inhibited food intake versus i.c.v. vehicle-treated mice (*p*<0.01 and *p*<0.001, respectively; Fig. 6A and B). Compared with vehicle, mice treated with NPS showed a significant reduction of food intake in the 0-0.5 h during 24 hours period (Fig. 6). However, during the second hour following treatment, the mice injected with NPS ate significantly more than the control (*p*<0.01; Fig. 6B).

### NPS induced c-Fos labeling in the olfactory cortex

**Figure 7:**
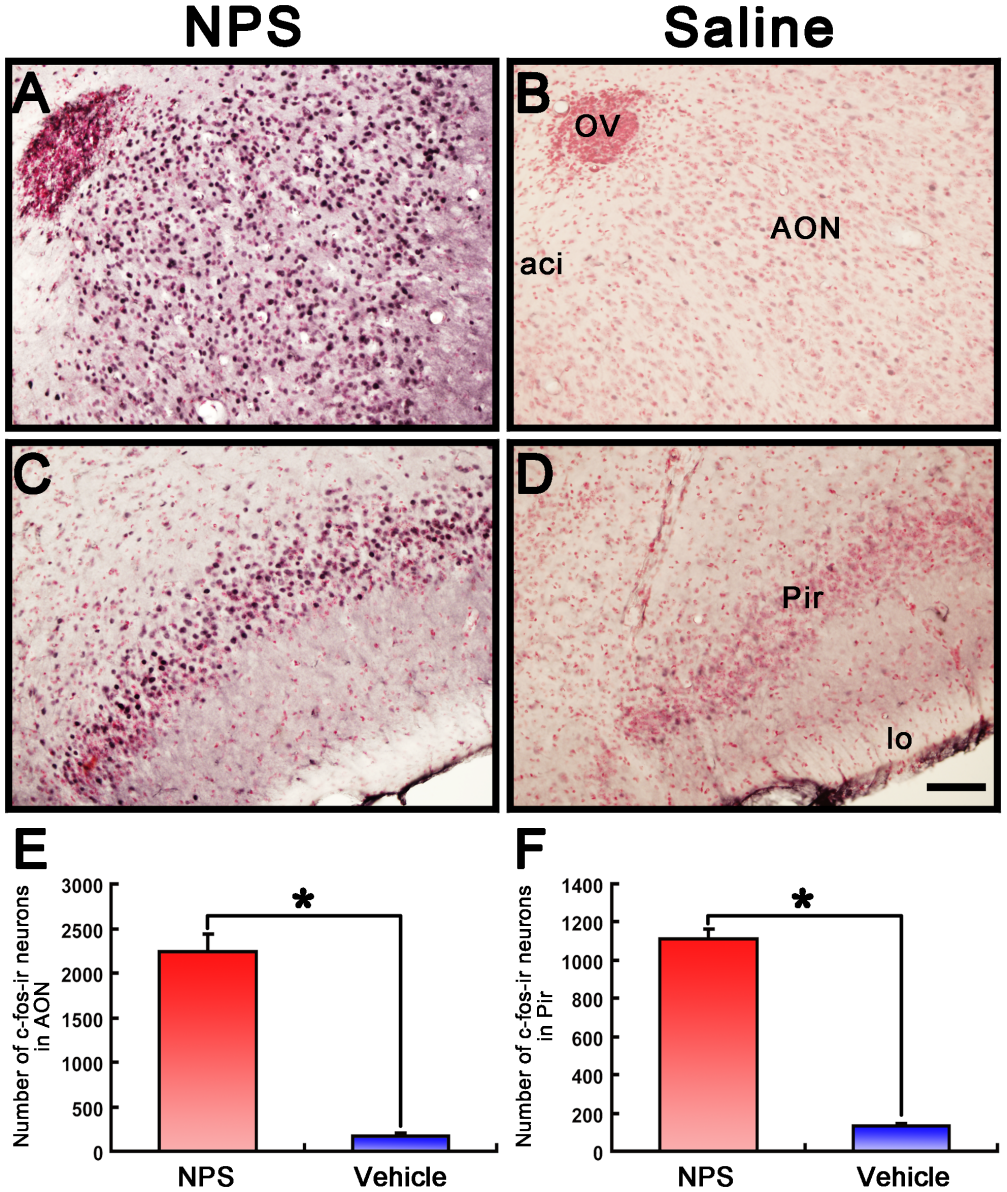
Effects of i.c.v. injection of NPS on Fos immunoreactivity in the AON and Pir in the mouse. **A-D** photomicrographs show Fos-ir neurons (black) in the AON and Pir in NPS- and vehicle-treated mice, respectively. **E, F.** Histograms show quantitative analysis of the number of Fos-ir neurons in the AON and Pir following NPS (n = 4 mice) and vehicle (n = 5 mice) i.c.v. injection. Values are means ± SEM. * *p*<0.001. Data were analyzed by independent student's t-test. Bar = 100 µm. Abbreviations: aci, anterior commissure, intrabulbar part; AON, anterior olfactory nucleus; lo, lateral olfactory tract; Pir, piriform cortex; OV, olfactory ventricle.

In the olfactory cortex, central administration of NPS (0.5 nmol) induced a large number of Fos-ir neurons in the AON (Fig. 7A) and Pir (Fig. 7C) and a moderate number of Fos-ir neurons in the VTT, the ACo and LEnt (Table. 1), and the numbers were significantly more than that seen in vehicle injection. In the present study, we focused on the AON and Pir areas because they are well-known to play a key role in olfactory function and regulation [28]. In comparison with vehicle-treated mice, NPS significantly increased the number of Fos-ir neurons by 13.2-fold (2236±199 vs. 169±15) in the AON (Fig. 7E) and 8.6-fold (1113±49 vs. 128±16) in the Pir (Fig. 7F). In addition, NPS also induced an increase in the number of Fos-ir neurons in the motor and somatosensory cortex, amygdala, periaqueductal gray, tuberomammillary nucleus, arcuate hypothalamic nucleus, and the perifornical nucleus and the lateral hypothalamic area (data not shown).

### NPS-induced Fos-ir neurons in the olfactory cortex expressed NPSR

**Figure 8:**
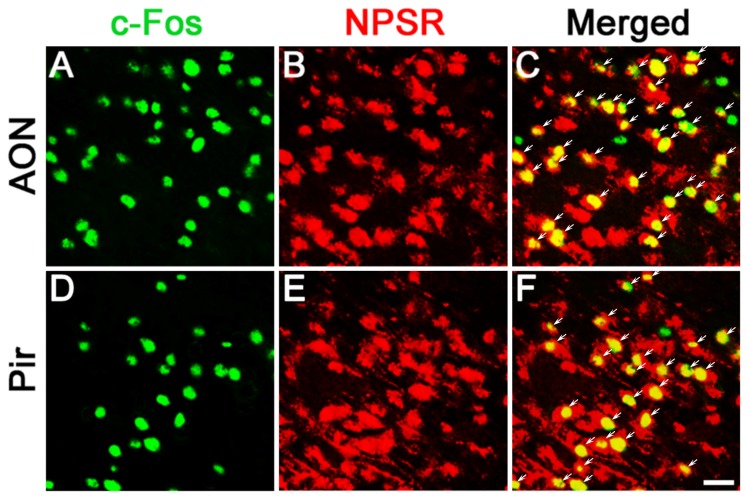
NPS-induced Fos-ir neurons bearing NPSR in the AON and Pir. Photomicrographs show Fos-ir neurons in the AON (A) and Pir (D) after NPS i.c.v. administration, NPSR-ir neurons in the AON (B) and Pir (E), and the co-expression of Fos-ir and NPSR-ir neurons in the AON (C) and Pir (F), respectively. Arrow (C and F) show the co-expression of Fos-ir and NPSR-ir neurons. Bar = 50 µm.

To determine whether the NPS-induced Fos-ir neurons in the AON and Pir express NPSR. Fos-ir staining combined with NPSR-ir staining were performed. As shown in Fig. 8, the percentage of Fos-ir neurons that also display staining for NPSR were 88.5±1.1% in the AON (Fig. 8A-C) and 98.1±0.4% in the Pir (Fig. 8D-F), respectively.

**Table 1:** Number of Fos-ir neurons in the VTT, ACo and LEnt after i.c.v. injection of NPS or vehicle.

	Number of Fos-ir neurons		
Groups	VTT	ACo	LEnt
NPS (n = 4)	297.00±13.22 *	384.00±11.58 *	1583.50±43.52 *
Vehicle (n = 5)	100.00±5.46	204.40±15.91	850.00±19.79

Values are expressed as means ± SEM. Cells were counted bilaterally per animal. * *p*<0.001, compared with vehicle-treated mice. Data were analyzed by independent student's t-test.

## Discussion

The present study firstly demonstrated that i.c.v. administration of NPS in mice facilitated the olfactory abilities by reducing the latency to find the buried food and increasing olfactory differentiation of different odors (Fig. 2–3). Among the three doses, 0.5 nmol of NPS would probably already activate maximally its brain targets, because in this dose, NPS reduced the latency most to find the buried food, and significantly promoted the dishabituation and habituation to all test odors. These results indicate that NPS could enhance the ability to smell volatile odors and to detect and differentiate the different odors. Several classical neurotransmitters derived from the brainstem region, for instance, the noradrenergic nucleus locus coeruleus [29]–[32] and the serotonergic raphe nucleus [33], have been shown to modulate olfactory behavior. In addition, several neuropeptides such as tachykinin-related peptides, short neuropeptide F and FMRFamide are also involved in the modulation of olfactory function [34]. Clark et al. reported that NPS is originated from the Kölliker-Fuse nucleus and pericoerulear area of the mouse brainstem and that NPSR mRNA is highly expressed in the olfactory cortex [2]. Taken together, NPS projections from the Kölliker-Fuse nucleus and pericoerulear area of the mouse brainstem to the olfactory cortex may provide mechanistic basis for its regulation of olfactory function.

Olfaction is of great importance to mammals' survival, and influences a variety of social activities, including recognition, mate selection, fear responses to predator odors and food intake, especially in rodents [23], [24]. In the present study, when 0.5 or 1 nmol of NPS were respectively i.c.v. administrated in fasted mice, food intake was dose-dependently reduced during the first half hour compared with vehicle-treated mice (Fig. 6). These findings are consistent with those of earlier observations in which NPS inhibits food intake in mice and rats [9], [35]–[37]. The mechanisms by which NPS regulates food intake are unknown so far. However, some reports described by Fedeli et al. and Peng et al. suggest that the paraventricular nucleus of the hypothalamus as well as NPSR are involved in the anorectic action of NPS [9], [36]. These results indicate that NPS enhances olfactory function but inhibits ingestion.

As shown in Figs. 3, NPS dose-dependently increased the total sniffing time during olfactory habituation and dishabituation tasks. Sniffing is typically assumed to be part of arousal behaviors [38]. During fast-wave state of the neocortical EEG, olfactory cortical neurons showed robust spike responses to adequate odorants, whereas they showed only weak responses during slow-wave state [39]–[41]. Our earlier work has demonstrated that NPS significantly increases wakefulness accompanied by an increase in EEG high frequency activities (14.5–60 Hz) and significantly decreases slow-wave sleep and paradoxical sleep in rats [7]. Therefore, sniffing behavior and the increased sniffing time are probably due to the increase of arousal, locomotion and exploration induced by NPS.

More importantly, our study also aims at identifying of the potential targets through which NPS facilitates olfactory function by examining of neurons expressing Fos, the product of the immediate early gene that is expressed in association with neuronal activation [42], [43]. Our results show that central administration of NPS induced an increase in the number of Fos-ir neurons in several regions of olfactory cortexes, including the AON, Pir (Fig. 7), VTT, ACo and LEnt (Table. 1). It is considered to occur in the AON for much of the initial odorant feature convergence involved in the early stages of building odor objects [28]. While the Pir, the largest region of olfactory cortex, would perform higher order associations between odor objects and hedonics, context and other odors [15]. Our present study was also designed to investigate whether the effects of NPS on the regulation of olfactory function were selectively antagonized by NPSR antagonist, and furthermore whether the neurons activated by NPS expressed its cognate receptor in the olfactory cortex. The results show that the effects of NPS on the regulation of olfactory function were blockaded by NPSR antagonist of [D-Val^5^]NPS (Fig. 4 and 5), and that the vast majority of the Fos-ir neurons activated by NPS in the AON and Pir also contained NPSR (Fig. 8C and F). A large number of literatures show that NPS selectively binds NPSR with high affinity to produce biological actions [1], [9], [10], [12], [44], [45]. Our results strongly suggest that NPS facilitates olfactory ability through activation of the neurons bearing NPSR in the olfactory cortex.

In summary, central administration of NPS in mice enhances olfactory functions by reducing the latency to find the buried food and increasing olfactory differentiation of the same and different odors. These effects are receptor-specific because they can be blocked by its selective antagonist NPSR [D-Val^5^]NPS. Furthermore, central administration of NPS markedly activates the c-Fos expression in the neurons of the olfactory cortex, most of which also express NPSR, indicating that NPS facilitates the olfactory functions through activation of the NPSR in the neurons of the olfactory cortex.
